# A study protocol evaluating the effectiveness of the older adult’s digital health literacy (ODDHeaL) intervention

**DOI:** 10.1371/journal.pone.0359396

**Published:** 2026-09-28

**Authors:** Nor Hazimah Rosle, Halimatus Sakdiah Minhat, Lim Poh Ying, Siti Aisah Mokhtar

**Affiliations:** Department of Community Health, Faculty of Medicine and Health Sciences, Universiti Putra Malaysia, Serdang, Selangor, Malaysia; Waseda University: Waseda Daigaku, JAPAN

## Abstract

Digital health literacy (DHL) is recognised as a vital determinant of healthy ageing, directly influencing the ability of older adults to navigate an increasingly technology-driven healthcare landscape. Despite increasing internet penetration, overall DHL among the elderly remains significantly low. Guided by the social cognitive theory (SCT), the ODDHeaL intervention module aims to improve the DHL of the community-dwelling older adults. A single-blinded, two-arm cluster randomised controlled trial was conducted to evaluate the Older Adults Digital Health Literacy (ODDHeaL) module. Individuals aged 55 years and older who own a smartphone with their own internet access, and, having normal M-ECAQ and Katz ADL scores, will be included in the study. The development of the ODDHeaL module or intervention was systematically guided by the ADDIE model, with the content developed based on an extensive literature review and input from experts from related fields. The newly developed module was tested across six clusters, which were randomly allocated to intervention and control groups and evaluated at baseline, immediately, and 2 months after the intervention. An intention‑to‑treat approach will be used during the analysis. This study may reveal the positive effects of the ODDHeaL intervention module on DHL among older adults, as well as health literacy, perception towards online health information, self-efficacy and health information-seeking behaviour (HISB). With improved DHL, older adults are expected to have better disease self-management by transforming them from passive recipients of care into active participants in their own health. This study was prospectively registered with the Thai Clinical Trials Registry (TCTR20250811007) on 11 August 2025, prior to participant enrolment.

## Introduction

The 21st century has witnessed a significant surge in digitalisation across various sectors, fundamentally altering how services are delivered and consumed [1]. Digital transformation is a key driver of the fourth industrial revolution, or better known as Industry 4.0, and it is becoming an indispensable requirement that affects all aspects of life in the current globalisation [2]. In an increasingly digitalised era, technology plays a pivotal role in enabling individuals to access and comprehend health information [3]. Digital health literacy (DHL) is a fundamental skill that individuals ought to have to access and utilise digital health information and services effectively [4].

The COVID-19 pandemic has undeniably accelerated the pace of digital transformation [5]. As physical distancing and lockdowns became the norm, digital solutions were rapidly deployed to ensure continuity in business, education, and particularly healthcare. The implementation of digital health interventions (DHI) like telemedicine has enabled the continuous delivery of healthcare services during the pandemic while protecting both patients and healthcare professionals from getting infected [6]. Additionally, DHIs have demonstrated significant benefits for older adults, such as promoting healthy ageing, facilitating continuous remote medical care [7,8] and promoting weight loss [9].

The WHO has adopted a Global Strategy on Digital Health (2020–2025), which emphasised the need for digital health technologies to promote healthy lives and well-being globally [10]. Aligned with the global strategy, Malaysia has proposed enhancing and integrating digital technologies into healthcare, as outlined in the recent Health White Paper [11].

Despite the considerable benefits of digitalisation, there remains a significant disparity in access, particularly among socially disadvantaged groups such as older adults [12,13], often referred to as the digital divide. If the disparity involves access to digital health services, it is called the digital health divide. Paradoxically, older adults are among populations that will benefit most from technologies, especially DHIs [14].

Nevertheless, research highlights a considerable percentage of the older adult population continues to be disconnected from the digital environment [15,16]. A study in Malaysia showed only that 32.3% older adults use the internet, despite 86.4% having internet access at home [17]. Unlike their younger counterparts, older adults face multifaceted barriers that their engagement with technology. Factors such as the increased prevalence of chronic diseases, lack of experience and access to technology, and other limiting factors limit the use of many older adults [18]. Other than that, low DHL levels among older adults is also one of the significant determinants of low adoption of DHIs on any digital health platforms (DHPs) among older adults [19,20].

A comprehensive systematic review conducted by Xie, Zhang [21], revealed that the DHL levels among older adults range from a minimum of 17.24 to a maximum of 32.9 on the eHealth Literacy Scale (eHEALS), which has a maximum score of 40. The eHEALS instrument, developed by [22], indicates that a higher score represents higher DHL. Disparities in DHL are further highlighted by regional studies; for instance, in Iran, where a score of 26 or above is considered indicative of high DHL, only 37% of older adults exhibited high DHL [23]. Similarly, a study conducted in Malaysia found that the mean eHEALS score among adults visiting primary health care facilities was only 24.4, suggesting that this population possesses low DHL. Furthermore, the study observed a significant decline in DHL with increasing age [24]. These findings underscore the pressing need for targeted interventions to enhance DHL among older adults.

Although DHL plays a crucial role in promoting the efficient utilisation of DHP, particularly among the senior population, DHL is affected by a wide range of factors [25,26]. A scoping review by Wang and Luan [27] identified various influencing factors categorised into sociodemographic aspects, digital instrument-related considerations, social dynamics, and psychological elements. Individual factors such as age [28–30], education level [31,32], marital status [33], household income [24], and employment status [34] have been shown to influence DHL. Additionally, health literacy and perception toward the internet were also found to significantly influence DHL [35,36]. Confidence in using digital devices was also found to influence DHL [37].

### Objectives

In view of the effectiveness of a theory-based intervention, this study aims to develop, validate and evaluate the Older Adults Digital Health Literacy (ODDHeaL) module/ intervention which was developed based on the social cognitive theory and guided by the ADDIE model.

### Hypothesis

It is hypothesised that the ODDHeaL module or intervention is effective in improving DHL, health literacy, perception towards online health information, self-efficacy and health information-seeking behaviour (HISB) among older adults, as evident by significant differences in scores between the intervention and control groups, as well as between different time points.

### Design

The trial is designed as a prospective parallel group cluster RCT to evaluate the effectiveness of the ODDHeaL intervention (intervention group) against the health education module unrelated to digital health (control group) at 3-time point comparisons. Six clusters of senior citizen activity centres were randomly allocated to the intervention and control group in a 1:1 ratio. The full trial protocol is provided in this manuscript. The statistical analysis plan is described within the methods section.

## Materials and methods

### Study setting

The study is conducted in registered senior citizen activity centres, also known as “Pusat Aktiviti Warga Emas” (PAWE), under the Social Welfare Department in Johor. The Social Welfare Department (SWD) functions under the jurisdiction of the Ministry of Women, Family, and Community Development.

Johor is a state situated at the southern tip of Peninsular Malaysia, with a population of 4.10 million in 2023, in which 456.9 thousand were those aged 60 and above, accounting for 11.1% of the total population [38]. It consists of 10 districts with Johor Bahru as the state. Approximately 94% of the households in Johor have access to the internet [39]. Despite having one of the highest public primary care clinics in Malaysia, with DHP Johor is one of the states with the lowest percentage of digital health usage in Malaysia [40,41].

### Eligibility criteria

The sampling population for this study is older adults who are registered members of the PAWEs located in Johor. Individuals who are aged 55 years and older, own a smartphone and have their own internet access, have normal cognitive function (M-ECAQ>4) and normal functional status (Katz ADLs > 4) will be included in the study.

### Intervention

The ODDHeaL module or intervention was developed based on an extensive literature review and from older adults and expert panels in related fields. ADDIE (Analysis, Design, Development, Implementation and Evaluation) model was used to systematically guide the development of the module which is summarised in Table 1.

**Table 1:** A systematic step-by-step procedure for intervention module development according to ADDIE model.

Phase	Module development and content
**Analysis**	Needs assessment was conducted to explore the learning requirements, barriers and preferences of older adults in using smartphones for health-related purposes. These were assessed through: • Extensive review of relevant literature on DHL among older adults, as well as behavioural theories that had been effectively applied in DHL related interventions. • Preliminary survey using open ended questions were conducted among 5–10 older adults at PAWEs in Johor, as well as public health experts and health educators in related fields (core functions of smartphones among the older adults, health aspects of interest to be learned via smartphones, methods of learning). • Insights obtained from this phase informed the scope and objectives of the module.
**Design**	• Clear learning objectives and structuring the content of the module was formulated based on findings obtained from the analysis phase. Module was structured according to: i. Basic smartphone skills ii. Searching skills for online health information iii. Perceptions of internet health info (reliability, usefulness, accuracy, credibility) iv. Self-efficacy in using smartphones for health v. General health literacy vi. Health information seeking behaviour (HISB) Instructional strategies were planned to include guided demonstrations, group discussions, practical exercises, and lectures. Instructional strategies were planned to include guided demonstrations, group discussions, practical exercises, and lectures
**Development**	Module materials, including PowerPoint slides, step-by-step handbooks, and mock scenarios, were developed based on the planned design. Content validation was conducted with four experts (public health and digital health engineering experts), and face validation was performed among health educators, graphic designers, and five older adults of similar background. The module content was revised based on feedback from the content and face validation.
**Implementation**	A pre-testing session was conducted at a PAWE in Johor to evaluate participant engagement and identify logistical issues. Participant engagement was assessed through observations and feedback on attendance, responses during sessions, session length, and perceived interest. The feedback obtained was used to refine the study procedures and intervention delivery before the main study.
**Evaluation**	The evaluation consists of both formative and summative assessments. Formative evaluation was conducted throughout the development and implementation phases. Summative evaluation assessed the intervention’s impact on outcomes using pre- and post-intervention surveys.

The intervention module consists of four sessions with details of content as outlined in Table 2. To promote intervention fidelity, all ODDHeaL sessions were delivered by the same researcher using the same standardized module, presentation materials, handbook, session structure, and planned activities across all intervention centres. The duration of each session was monitored to ensure consistency with the planned intervention schedule, and the same intervention content and activities were delivered at each centre. Participants continue to receive their usual medical care during the study, and no restrictions were imposed on concomitant treatments.

**Table 2:** Content of ODDHeaL education module.

Session (duration)	Aim/Objective	Description of Activities	Variable	SCT Construct
**Session 1: Meet and connect** **(15 minutes)**	To help participants and the facilitator build rapport and trust. To introduce the module structure.	Participants take turns passing a stress ball and introducing themselves. They share their name, place of origin, and experience using technology. The facilitator explains the module objectives and structure using a PowerPoint presentation.	NA	NA
**Session 2: What is Digital Health Literacy?** **(1 hour)**	To help participants understand health, digital health, and DHL. To encourage positive perceptions of online health information. To help participants recognise and appreciate social support in their lives.	The facilitator gives a lecture using PowerPoint slides to explain digital health and its benefits. Participants take part in a group discussion to share their thoughts and correct misconceptions about online health information Participants draw a “Support Tree” to identify family, friends, and people around them who support them in using technology.	DHL Perception on online health information	Behavioral Capability Outcome Expectation
**Session 3: Building Confidence in Digital Skills** **(3 hours)**	To improve participants’ basic skills in using smartphones. To help participants feel confident, safe and responsible in using smartphone.	The facilitator demonstrates how to use smartphone features such as adjusting settings, connecting to Wi-Fi, and managing apps. Participants practice these skills on their own while group leaders assist those who need help. Participants complete small digital tasks step by step to build their confidence Participants were taught how to search and evaluate websites and online health information.	DHL Self-efficacy in using smartphone HISB Perception on online health information	Self-Efficacy Observational Learning
**Session 4: Navigating Health Through Digital Platforms** **(1.5 hours)**	To help participants find, evaluate, and use health information online. To improve decision-making about health using digital tools.	Participants work in groups to solve scenario-based health-related problems using their smartphones. They discuss their answers with the group and present their findings. The facilitator provides feedback and guides participants on how to verify reliable health information online.	Health Literacy Self-efficacy in using smartphone HISB	Behavioral Capability Self-Regulation Outcome Expectation

### Outcomes

The primary outcome is DHL, which refers to the perceived ability to search for, find, understand, evaluate, and apply health information obtained from electronic sources to address health-related needs [22]. It will be measured using eHEALS, with higher score indicating higher DHL. The secondary outcomes are:

i. Perception towards online health information refers to an individual’s evaluation of the reliability, usefulness, accuracy, up-to-dateness and concreteness of health information obtained from the internet [42]. It will be measured using a 5-point Likert scale.
ii. Health literacy refers to the ability of individuals to gain access to, understand, and use information in ways which promote and maintain good health for themselves, their families, and their communities [43,44]. It is measured using the HLS-M-Q18 [45] using a 4-point Likert scale with scores ranging from 0 to 50. The higher the health literacy score, the higher the health literacy of the participants [46].
iii. Digital self-efficacy refers to an individual’s perceived belief in their ability to use a smartphone effectively for digital and health-related tasks [47]. It is measured using the adapted version of the digital self-efficacy scale developed by Ulfert-Blank and Schmidt [47]
iv. Health information seeking behaviour (HISB) refers to the frequency of engaging in health information-seeking activities [48]. Responses are rated on a five-point Likert scale ranging from never to always. Higher scores indicate more frequent health information-seeking behaviour, while lower scores indicate less frequent or minimal health information-seeking behaviour.

All outcomes will be measured at baseline, immediately after the intervention exposure, as well as at 2 months follow up.

### Research flow and participant timeline

Recruitment was carried out in collaboration with PAWE supervisors and committee members, who assisted in identifying and contacting eligible members. Information sessions were conducted, and engagement with PAWE leadership helped encourage participation. Participants may withdraw from the study at any time without consequence. Participation may also be discontinued if a participant is unable to attend intervention sessions or experiences discomfort or distress related to study activities. The schedule of enrolment, randomisation, allocation to intervention and comparator arms, and outcome assessments at baseline, immediately post-intervention, and at 2 months post-intervention is presented as shown in Fig 1.

**Fig 1:**
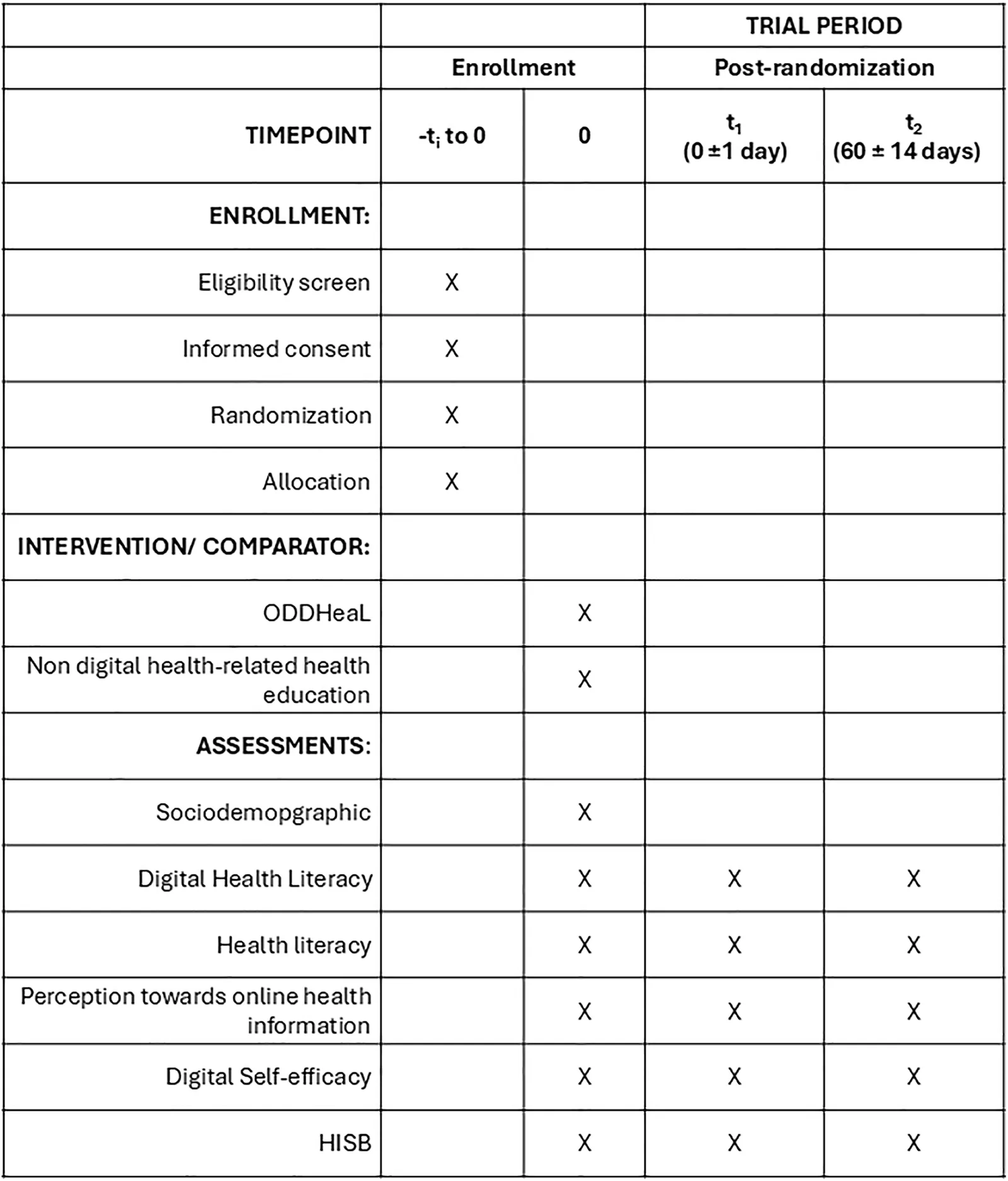
Flow diagram of study conduct based on CONSORT statement.

The SPIRIT 2025 checklist (**S**tandard **P**rotocol **I**tems: **R**ecommendations for **I**nterventional **T**rials) is also provided as supplementary document to defines the minimum standard content required in the clinical trial protocol. The checklist was developed in parallel with CONSORT 2025 (the reporting framework for final trial results) to modernize protocol standards, reflect contemporary research practices, and harmonize trial reporting (S1 Table).

### Sample size

The sample size was estimated using eHEALS data at 3 weeks post-intervention from a study by Nahm and Zhu [49], which reported DHL mean scores of 28.44 ± 6.16 in the control group and 31.58 ± 5.21 in the intervention group. The mean difference between groups was used to calculate the required sample size for comparing two independent means, assuming equal standard deviations, a 95% confidence level (α = 0.05), and 80% power (β = 0.20). This yielded a required total sample size of 50 participants prior to adjustment for clustering.

As the present study employs a cluster-randomised design, the sample size was adjusted using a design effect. An intracluster correlation coefficient (ICC) of 0.05 was assumed based on a previous cluster-randomised controlled trial involving older adults in Malaysia, which similarly used an ICC of 0.05 for its sample-size calculation [50]. Assuming an average cluster size of 20 participants, the design effect was calculated using the formula: design effect = 1 + ρ(m − 1), resulting in a design effect of 1.95. After adjustment for clustering, the required sample size increased to 98 participants.

To account for an anticipated attrition rate of 20%, the sample size was further inflated, resulting in a final required sample of 118 participants. Therefore, 59 participants will be allocated to each study arm. Because the sample size calculation was specifically powered for the primary outcome (digital health literacy), all statistical analyses for secondary outcomes will be considered exploratory.

### Sampling

A cluster random sampling approach was used, with PAWEs serving as the sampling units. There are 22 PAWE in Johor, from which six clusters were selected based on the required sample size and estimated cluster size.

Each PAWE is estimated to have approximately 20 active registered members. Given the total required sample size of 118 participants, the number of clusters needed was determined by dividing the total sample size by the estimated number of participants per cluster, resulting in six clusters. These were equally allocated, with three clusters assigned to each study arm.

The six centres were selected using a simple random procedure from the full list of centres. Lists of active members were then be obtained from the selected centres, and all eligible members were invited to participate in the study.

### Blinding and allocation concealment

Allocation was carried out in a 1:1 ratio using an online random pair generator [51], with three PAWEs allocated into the intervention group, and the other three into the control group. All eligible participants within each PAWE received the same group allocation. Single-blinding methods was utilised, in which participants were unaware of their assigned group. Blinding the field researcher was not feasible, as the researcher was responsible for delivering the intervention module to the selected participants.

To ensure concealment of allocation, randomization of group allocation was done after the recruitment of all eligible participants. The group allocation is strictly confidential and only known to the researcher. Throughout the study, participants received uniform communication and instructions regardless of their assignment group to maintain blinding integrity. Unique identifiers were assigned to maintain anonymity and prevent accidental disclosure of group assignments during data collection. Unblinding of participant allocation will only occur if required for safety reasons, and the decision will be made by the principal investigator. Any unblinding will be documented.

### Data collection and management

Participation was voluntary, and participants may withdraw at any time without consequence. Following prospective trial registration with the Thai Clinical Trials Registry (TCTR20250811007) on 11 August 2025, participant recruitment commenced in November 2025 and was completed in July 2026. Data entry, verification, and analysis are currently underway, with overall study results anticipated by March 2027.

Potential participants were initially screened for eligibility using the Malay Elderly Cognitive Assessment Questionnaire (M-ECAQ), the Katz Index of Independence in Activities of Daily Living (ADL), and confirmation of smartphone ownership with internet access. Older adults who meet the inclusion criteria and provide verbal and written informed consent were enrolled in the study. Written informed consent was obtained from all participants by the researcher before enrolment, after explaining the study and an information sheet.

Data was collected using structured, self-administered questionnaires at three timepoints: baseline (T0), immediately after the intervention (T1), and two months after the intervention (T2). The baseline data (T0) was collected from both the intervention and control groups using a valid and reliable self-administered questionnaire. The intervention group received the ODDHeaL intervention module over two separate days at their respective PAWEs. Meanwhile, the control group received a health education module unrelated to DHL.

Participants in the control group received a 2-hour health education module on mental health among older adults, delivered by the same researcher using similar instructional strategies as the intervention arm, including lectures, group activities, and individual activities. The control module did not include any digital health literacy content. The total contact time was 2 hours compared with approximately 5 hours and 45 minutes in the intervention arm, where additional time was required for step-by-step instruction and hands-on practice of digital health skills. This comparator was chosen to provide a structured educational activity for the control group, allowing the specific effects of the DHL intervention to be evaluated. The study instruments have undergone face validity and reliability assessment prior to use. Data collection form is available from the corresponding author upon reasonable request.

Participants received standardized instructions and completed forms were checked immediately for completeness to ensure data quality. Any incomplete responses were addressed on the spot by guiding participants to complete unanswered items. Participants continued their usual daily activities and medical care during the study. No restrictions were imposed on concomitant activities or treatments. Participant retention was supported through WhatsApp reminders and collaboration with PAWE supervisors and committee members. Attrition and missing data were documented at each timepoint.

All study data will be entered by the researcher into an electronic dataset and coded using participant identification numbers without personal identifiers. Completed questionnaires will be checked for completeness before entry to promote data quality. Data will be stored securely, with access limited to the research team.

A formal Data Monitoring Committee was established because this study involves a minimal-risk educational intervention. No interim analyses or stopping guidelines were planned. Study conduct was overseen by the research team in accordance with the institutional ethics approval.

### Statistical analysis

The collected data will be analysed using IBM Statistical Package for Social Science (SPSS) version 29, which will involve both descriptive and inferential statistics. Data exploration will be conducted to detect errors in data entry, missing data, and the presence of outliers. Descriptive statistics will be presented as means and standard deviations (SD) for normally distributed continuous variables, medians and interquartile ranges (IQR) for non-normally distributed continuous variables, and frequencies and percentages for categorical variables. Normality of continuous variables will be assessed using a combination of graphical and numerical methods. Histograms and Q-Q plots will be examined together with measures of skewness and kurtosis, and the overall distributional pattern will be considered rather than relying on a single normality test.

Generalised Estimating Equations (GEE) will be utilised to examine main effects and interactions within and between the intervention and control groups across different time points (baseline, immediately post-intervention, and two months post-intervention). For continuous outcomes, models will be fitted using a normal distribution with an identity link function and an unstructured working correlation structure. For binary outcomes, models will be fitted using a binomial distribution with a logit link function and an unstructured working correlation structure.

The primary analysis will follow the intention-to-treat principle. Missing data will first be assessed for their pattern and mechanism and will be assumed to be missing completely at random (MCAR). Multiple imputation using predictive mean matching will be used to handle missing values. Baseline variables will be screened in preliminary analyses as potential confounders, and variables showing a statistically significant association at p < 0.05 will be included in the adjusted GEE model. Where multiple pairwise comparisons are performed, an appropriate adjustment for multiple testing will be applied.

### Ethical considerations

Ethical approval was obtained from the Ethics Committee for Research Involving Human Subjects, Universiti Putra Malaysia (JKEUPM) (JKEUPM Reference Number: JKEUPM-2024–680) prior to the commencement of the study. Verbal and written consent will be obtained from all participants before recruitment. The study was prospectively registered with the Thai Clinical Trials Registry (TCTR20250811007) on 11 August 2025, prior to the commencement of participant enrolment in November 2025. The study was also registered with the National Medical Research Registry (NMRR) (NMRR Reference Number: NMRR ID-24–03416-VX4), and permission was granted by the Social Welfare Department.

Participant privacy will be protected, and all survey responses will remain confidential in accordance with institutional and national research regulations. No datasets were generated or analysed for this study protocol. Data generated from the trial will be addressed in accordance with the applicable ethical requirements and data availability policy when the study findings are reported.

As this is a low-risk educational intervention, no significant harm was anticipated. Potential minor discomforts, such as fatigue or frustration during learning activities, could have occurred. Harms were assessed through participant self-report and observation by the researcher during sessions, and participants were allowed to withdraw at any time if they experience any discomfort. This manuscript reports the original protocol version 1.0 (finalized on 4^th^ September 2024), which has remained unchanged since participant recruitment began in November 2025. Any important protocol amendments will be subject to approval by the ethics committee, updating of the trial registry, and communication to relevant parties.

## Discussion

DHL has been recognised as a fundamental competency for the elderly, directly determining their ability to navigate a healthcare landscape that has increasingly shifted to digital-first delivery. Improving DHL among older adults involves the identification of its determinants and tackling these determinants through the implementation of effective interventions. DHL has been reported to be influenced by behavioural factors such as digital skill and competency [35], health information-seeking behaviour [34], self-efficacy [37], perception towards online health information [33] and health literacy [35,52]. Good support from family and having good social networks were also found to influence DHL among older adults [37,53]. In order to ensure efficacy, interventions designed to improve DHL must take into account each of these factors [54].

Previous studies have demonstrated that interventions targeting the improvement of older adults’ DHL have been found to have many positive benefits across several domains, including physical, behavioural, psychosocial, and cognitive aspects [21,27,55]. Lin, Ganji [56] conducted a study revealing that DHL exerts a significant direct and indirect influence on the medication adherence and quality of life (QoL) of older adults. Moreover, among Chinese older adults, DHL was shown to have a positive association with health-related quality of life (HRQoL) [B(SE) = 0.210(0.029), p < 0.001] [36]. High DHL was also significantly correlated with key components of healthy ageing, such as the absence of morbidity, functional limitations, depressive symptoms, and social isolation [55]. Furthermore, DHL was found to be positively associated with older adults’ mobility, self-care, and their usual activities [57]. These findings indicate that DHL played an essential part in contributing to the well-being and overall health of older adults, thus highlighting the need to enhance DHL within this particular age demographic.

Recognizing the critical need to enhance health outcomes through digital technologies, the World Health Organization’s (WHO) Global Strategy on Digital Health 2020–2025 underscores the importance of promoting the appropriate use of digital technologies for health. A key initiative in advocating for people-centred health systems, as outlined in this strategy, involves improving DHL [10]. Similarly, Malaysia’s Health White Paper on health system reform emphasised a commitment in prioritising digital health technologies to enhance data collection, analysis, and monitoring [11]. The successful implementation of the health reform is reliant upon improving the level of DHL, particularly among the older adult population, which represents the age group with the highest utilisation of healthcare services in Malaysia [58]. Thus, in line with these global and national directives, it becomes essential to prioritise interventions aimed at improving DHL.

Various interventions aiming to enhance DHL among older adults have been proposed, as evidenced by studies conducted by several researchers [21,27,59,60]. Most of these interventions, however, were single-group, pre-test, and post-test interventions [27,54,59]. While these studies do demonstrate improvement in DHL [54], it has long been recognised that randomised control trials (RCTs) yield better results in health-related research by carefully eliminating possible biases [60]. Therefore, adopting randomised control trials is not only in line with a larger push for evidence-based practices, but it also improves the level of scientific sophistication needed to reliably and effectively enhance the interventions that aim to improve older adults’ DHL.

Initially, most of the interventions of DHL do not use any theoretical framework [60], but in a later systematic review, more interventions were found to have a theory-based approach [59]. The meta-analysis by Dong et al. in 2023 added to the evidence of this new finding, revealing that interventions using a theory-based approach showed greater effectiveness in improving eHealth literacy [54].

Nevertheless, most of the interventions were only focusing on self-efficacy theory and lacked inclusion of environmental component, which is an essential factor influencing DHL among older adults [27,59]. Social Cognitive Theory (SCT) is a health behavioural model that evolves from social learning and self-efficacy theory [61,62]. The factors influencing DHL, as mentioned previously are able to explain the dynamic interplay of personal, environmental and behavioural factors in SCT, therefore SCT will be employed in the development of this intervention module.

The study findings will be disseminated through publication in peer-reviewed journals, conference presentations, and reporting in the trial registry. The findings will also be communicated to relevant public health and ageing-care stakeholders.

### Limitation

Several limitations of this study protocol warrant consideration. Blinding of the researcher who delivered the intervention was not feasible, which may introduce performance bias. Although intervention delivery was standardised across centres using the same researcher, materials, session structure, duration, and activities, no formal intervention fidelity checklist or independent fidelity assessment was conducted. The intervention and control groups also had unequal contact time, with approximately 5 hours and 45 minutes of educational exposure in the intervention arm compared with 2 hours in the control arm, which may have influenced participant engagement and attention. The study involved only six PAWE clusters and included older adults who owned a smartphone with internet access and had adequate cognitive and functional status, which may limit the generalisability of the findings to other older populations. In addition, the intervention and study instruments were delivered in Malay and English, which may limit applicability to older adults who are less proficient in Malay and English or who primarily communicate in other languages. Finally, the study was powered based on the primary outcome of digital health literacy; therefore, analyses of the secondary outcomes should be interpreted as exploratory.

## Supporting information

S1 Table SPIRIT 2025 checklist. (DOCX)
